# Seroprotective Antibodies to 2011 Variant Influenza A(H3N2v) and Seasonal Influenza A(H3N2) among Three Age Groups of US Department of Defense Service Members

**DOI:** 10.1371/journal.pone.0121037

**Published:** 2015-03-27

**Authors:** Jennifer M. Radin, Anthony W. Hawksworth, Ryan G. Ortiguerra, Gary T. Brice

**Affiliations:** Operational Infectious Diseases Department, Naval Health Research Center, San Diego, California, United States of America; Centers for Disease Control and Prevention, UNITED STATES

## Abstract

**Background:**

In 2011, a new variant of influenza A(H3N2) emerged that contained a recombination of genes from swine H3N2 viruses and the matrix (M) gene of influenza A(H1N1)pdm09 virus. New combinations and variants of pre-existing influenza viruses are worrisome if there is low or nonexistent immunity in a population, which increases chances for an outbreak or pandemic.

**Methods:**

Sera collected in 2011 were obtained from US Department of Defense service members in three age groups: 19–21 years, 32–33 years, and 47–48 years. Pre- and post-vaccination samples were available for the youngest age group, and postvaccination samples for the two older groups. Specimens were tested using microneutralization assays for antibody titers against H3N2v (A/Indiana/10/2011) and seasonal H3N2 virus (A/Perth/16/2009).

**Results:**

The youngest age group had significantly (p<0.05) higher geometric mean titers for H3N2v with 165 (95% confidence interval [CI]: 105–225) compared with the two older groups, aged 32–33 and 47–48 years, who had geometric mean titers of 68 (95% CI: 55–82) and 46 (95% CI: 24–65), respectively. Similarly, the youngest age group also had the highest geometric mean titers for seasonal H3N2. In the youngest age group, the proportion of patients who seroconverted after vaccination was 12% for H3N2v and 27% for seasonal H3N2.

**Discussion:**

Our results were similar to previous studies that found highest seroprotection among young adults and decreasing titers among older adults. The proportion of 19- to 21-year-olds who seroconverted after seasonal vaccination was low and similar to previous findings. Improving our understanding of H3N2v immunity among different age groups in the United States can help inform vaccination plans if H3N2v becomes more transmissible in the future.

## Introduction

A new influenza A(H3N2) variant virus emerged in 2011, referred to as H3N2v, which contained a recombination of genes from swine H3N2 viruses and the M or matrix protein from the influenza A(H1N1)pdm09 (pH1N1) virus [1]. From 2005 to 2011, human infections with swine influenza viruses were relatively rare in the United States, with only 35 known cases [2]; however, the frequency of human cases increased dramatically in 2011 and 2012, when over 300 cases of H3N2v were identified [3,4]. These cases occurred predominately among young children exposed to swine at agricultural fairs [4]. Although there have only been a few suspected human-to-human transmitted cases [3,4], there is concern that this virus could mutate, improving its ability to spread between humans, causing new outbreaks or even a pandemic in the future. This is especially worrisome because H3N2 seasonal viruses have historically resulted in more severe illness, such as hospitalization and death [5,6].

Several studies have found that protective antibodies against H3N2v generally tend to increase among older age groups of children, peak among young adults, and then decrease among older age groups of adults [7–9]. These studies have also shown little to no immunity among children younger than age 10 years, which coincides with the ages of the H3N2v cases [7–9]. Additionally, several studies have found that vaccination with seasonal trivalent inactivated influenza vaccine (TIV) results in only small improvements in cross-protective immunity to H3N2v [7,9,10]. However, incongruous age categories, vaccine type used, different populations, small sample sizes, and different testing methods make comparisons between existing studies challenging.

To date, there have only been a few serologic studies assessing cross-protective immunity and seroconversion against H3N2v, and the one study completed in the United States used a broad age category for adults, which ranged from 18 to 49 years. Our study aims to add to the existing literature by evaluating the cross-protective antibodies among more-specific adult age categories of US Department of Defense (DoD) service members in 2011. Military-specific populations are especially important because they are often at increased risk of respiratory infections due to high-density living quarters and physical demands. Gaining a better understanding of pre- and post- vaccine immunity will help inform targeted immunization or treatment of high-risk groups if an H3N2v outbreak or pandemic were to emerge.

## Materials and Methods

### Participants and procedures

Three age groups of active-duty DoD service members were used for this study: 19–21 years, 32–33 years, and 47–48 years. Pre- and post-vaccination sera were obtained from the youngest group (all were US military basic trainees at either Coast Guard Training Center, Cape May, New Jersey; Fort Jackson, South Carolina; or Marine Corps Recruit Depot Parris Island, South Carolina) during a public health investigation, and sera for the other two groups were obtained from the DoD Serum Repository. All serum samples were de-identified for this study. Forty-nine paired sera samples were tested for the youngest age group, and 50 single serum samples were tested for each of the two older age groups.

Pre-vaccination sera for the 19–21 year olds were collected between August 2009 and January 2011 and post-sera were collected in March 2011. More than 90% of DoD service members receive annual seasonal influenza vaccines, therefore the two oldest age groups were considered vaccinated. Post-vaccination sera for the older two groups were collected in January 2011, about 2 to 4 months after vaccination. All sera were collected prior to the first cases of H3N2v identified in the US in August 2011 [3]. Both TIV and LAIV vaccines were administered and for the 2010–2011 influenza season, the influenza vaccine composition consisted of an A/California/7/2009 (H1N1)-like virus; an A/Perth/16/2009 (H3N2)-like virus; and a B/Brisbane/60/2008-like virus [11].

Specimens were tested using microneutralization (MN) assays for antibody titers against H3N2v (A/Indiana/10/2011) and seasonal H3N2 virus (A/Perth/16/2009). Viruses used were grown in Madin-Darby canine kidney cells, and adjusted to a consistent concentration throughout the MN assays. Each specimen was tested in duplicate, and the geometric mean of both was used. An MN titer of ≥80 was considered seropositive [7,9], and a 4-fold or greater rise in titer from pre- to post-vaccination with a postvaccination MN titer of ≥80 was considered seroconversion.

### Statistical analysis

The mean of the geometric mean, as well as the percentage of specimens with MN titers ≥80 were calculated for each age group. An analysis of variance (ANOVA) was used to identify if any differences in the means existed across the age groups, and Tukey’s honestly significant difference post hoc test was used to identify where the differences existed. A chi-squared test was used to identify any differences in the percentage of MN titers ≥80 across age groups.

All statistical analyses were conducted using SAS software, version 9.3 (SAS Institute Inc., Cary, North Carolina). PROC FREQ and PROC MEAN were used for frequencies and means, respectively, and PROC GLM was used for the ANOVA and post hoc tests. Additionally PROC CORR was used to calculate the Pearson correlation coefficient comparing the geometric mean titers of H3Nv and seasonal H3N2.

### Ethics statement

This research was conducted in compliance with all applicable federal and international regulations governing the protection of human subjects in research (Protocol NHRC.2013.0025). Authors were involved in collection of sera samples from the youngest age group and had access to personally identifiable information (PII) prior to its anonymization for the study. For the older two age groups, authors were not involved with sera collection and never had access to PII, although it is stored in a DoD sera repository. Since all specimens in this study were collected previously and were de-identified for the purposes of this study, the Naval Health Research Center institutional review board committee classified this study as minimal risk, exempt from full committee review.

## Results

**Fig 1:**
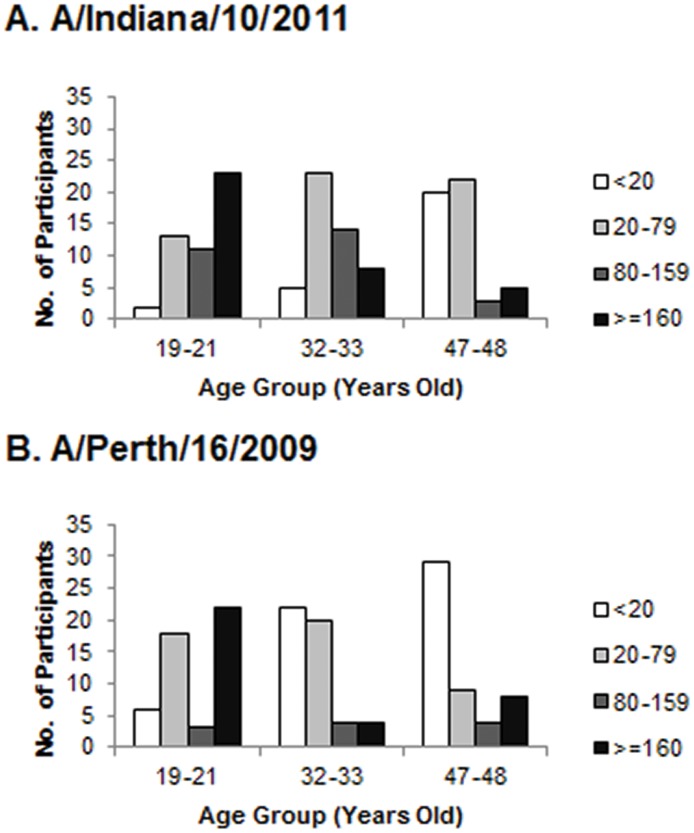
Postvaccination microneutralization titers by three age groups of US Department of Defense service members. (A) H3N2v (A/Indiana/10/2011) and (B) seasonal H3N2 (A/Perth/16/2009).

Postvaccination MN titers against H3N2v differed across the three age groups. The youngest age group (19-to-21-year-olds) had the highest geometric mean titer of 165 (95% confidence interval [CI]: 105–225), and the two older age groups, aged 32–33 and 47–48 years, had the lowest geometric mean titers, with 68 (95% CI: 55–82) and 46 (95% CI: 24–65), respectively. The youngest age group was statistically higher than the older two (p<0.05) in terms of geometric mean titers, but the two older groups were not significantly different from each other. The proportion with mean postvaccination titers ≥80 for H3N2v was 69% for those aged 19–21 years, and declined to 44% and 16% among those aged 32–33 and 47–48 years, respectively. All age groups were significantly different from each other for the proportion with mean titers ≥80 (p<0.05). Additionally, as the ages increased, there was a steady decline in the number of participants with titers ≥160 and a steady increase in the number of participants with titers ≤20 (Table 1 and Fig. 1).

**Table 1:** Geometric mean titer, percentage with microneutralization titer ≥80 or hemagglutination inhibition titer ≥40, and seroconversion, by age group and selected summary of previous studies with similar age groups.

Study population	Age group (years)	Birth years	n	Vaccination: LAIV, TIV, Both (% LAIV), or cross-sectional sample	Antigen	Test type	Geometric mean MN or HI titer (95% CI)		% with MN titer ≥80 or HI titer ≥40		Sero-conversion (%)
							Pre-vaccination	Post-vaccination	Pre-vaccination	Post- vaccination	
US (DoD)	19–21	1990–1992	49	Both (77%)	H3N2v^b^	MN	124 (86–162)	165 (105–225)	55 (41–69)	69 (56–82)	12 (3–21)^a^
					H3N2^c^		69 (39–100)	175 (97–253)	29 (16–42)	51 (37–65)	27 (15–39)^a^
	32–33	1977–1978	50	Both (38%)	H3N2v^b^	MN		68 (55–82)		44 (30–58)	
					H3N2^c^			38 (22–54)		16 (6–26)	
	47–48	1962–1963	50	Both (45%)	H3N2v^b^	MN		46 (24–68)		16 (6–26)	
					H3N2^c^			52 (28–76)		24 (12–36)	
US (NHANES & a 2010–11 TIV study)	18–49		30	TIV	H3N2v^d^	MN	55 (31–98)	95 (51–177)	43	63	13
					H3N2^c^		31 (16–61)	172 (94–316)	27	70	50
Canada	20–59		65	TIV	H3N2v^b^	HI	14 (10–18)	22 (16–29)	26 (15–37)	38 (26–50)	11 (3–19)
					H3N2^e^		22 (16–29)	128 (94–173)	35 (23–47)	89 (81–97)	65 (53–77)
							Cross-sectional sample		Cross-sectional sample		
	20–29		98	Cross-sectional	H3N2v^b^	HI	43 (34–54)		59 (49–69)		
					H3N2^f^		16 (12–22)		28 (19–36)		
	30–39		100	Cross-sectional	H3N2v^b^	HI	22 (18–26)		35 (26–44)		
					H3N2^f^		15 (11–18)		31 (22–40)		
	40–49		100	Cross-sectional	H3N2v^b^	HI	9 (8–10)		7 (2–12)		
					H3N2^f^		12 (9–15)		25 (17–34)		
Norway	18–24	1993–1987	28	Cross-sectional	H3N2v^g^	HI	37 (27–52)		71 (53–86)		
	25–34	1986–1977	45		H3N2v^g^		41 (31–53)		71 (57–83)		
	35–44	1976–1967	27		H3N2v^g^		27 (20–35)		48 (30–67)		
	45–54	1966–1957	22		H3N2v^g^		11 (8–16)		14 (4–33)		

DoD, Department of Defense; HI, hemagglutination inhibition; LAIV, live attenuated influenza vaccine; MN, microneutralization; NHANES, National Health and Nutrition Examination Survey; TIV, trivalent influenza vaccine.

^a^4-fold or greater increase, with postvaccination MN titers ≥80.

^b^A/Indiana/10/2011.

^c^A/Perth/16/2009.

^d^A/Minnesota/11/2010.

^e^A/Wisconsin/15/2009.

^f^A/Brisbane/10/2007.

^g^A/Indiana/08/2011.

For seasonal H3N2 titers, the youngest age group also had the highest geometric mean titer with 175 (95% CI: 97–253), followed by the oldest age group with 52 (95% CI: 28–76). The lowest titers were found in the middle age group with 38 (95% CI: 22–54), although they were not significantly different from the oldest age group. Similarly, the proportion with postvaccination MN titers ≥80 for seasonal H3N2 was 51% for those aged 19–21 years, 16% for 32–33 years, and 24% for 47–48 years. Interestingly, the titers for those aged 32–33 years were significantly lower for H3N2 than for H3N2v. The titer trends for seasonal H3N2 were more variable, with the youngest having very few intermediate titers of 80–159 (see Table 1 and Fig. 1).

In the youngest age group, 12% seroconverted to H3N2v and 27% seroconverted to seasonal H3N2 following vaccination (Table 1).

The Pearson correlation between H3N2v and seasonal H3N2 geometric mean titers was 0.65 (p<0.0001) for pre-vaccination among the 19- to 21-year olds and 0.59 (p<0.0001) for post-vaccination among all age groups.

## Discussion

H3N2v first emerged in 2011 with 13 cases identified between August 2011 and April 2012 [12], and it quickly grew to 305 between July 9 and September 7, 2012 [4]. However, modeling suggests that the actual number was much higher: approximately 2,000 cases during the first time period due to lack of identification by existing surveillance systems [12]. Among the 13 initial cases, 12 were children, with most under age 10 years, and 54% had known swine exposure [3]. Among the 305 cases, 92% were younger than 18 years, and 95% had direct or indirect exposure to swine [4]. Although most cases had swine exposure, there were 15 possible human-to-human transmitted cases [4]. The possibility of future human-to-human H3N2v transmission is a concern considering the high susceptibility among children and older adults.

In this study we found that young adults aged 19–21 years had significantly higher geometric mean titers (p<0.05) mean antibody titers against H3N2v and seasonal H3N2 compared with the older adults, aged 32–33 and 47–48 years. We also found that with increased age came decreased antibodies to H3N2v, although the geometric mean titers of the two older age groups in our study were not significantly different. Seropositivity to influenza may vary by age as a result of different exposures to circulating influenza viruses during childhood and different exposures in adulthood that would have boosted antibodies, thus leading to cohort effects [7]. Regional differences in circulating strains across countries may also influence seropositivity and explain some of the variation seen across study populations.

Due to the characteristics of our study population, it is unlikely that the participants were exposed to H3N2v directly. However, it is possible that this population, especially the youngest age group, had recent natural exposure to seasonal H3N2. Natural infection has been found to result in stronger immunologic responses than vaccination, especially in younger adults [13], which may explain the large proportion of participants in the youngest age group with seasonal H3N2 titers ≥160 (Fig. 1).

Similar seropositive proportions to H3N2v by age group have been found in other studies in the United States, Norway, and Canada [7–9]. The Canadian study used hemagglutination inhibition (HI) assay titers and found that 0% of children <5 years of age had seropositive antibodies for H3N2v, <20% of individuals aged ≤14 and ≥40 years, and approximately 50% for individuals aged 15–39 years [7]. The Norwegian study found 71% seropostivity among 18- to 34-year-olds, and the lowest seropositiivty among children younger than age 12 (0%) and 45- to 54-year-olds (14%) [8]. The previous US study found no seropositivity among children aged 6–35 months, 33% among adults aged 18–49 years, and 17% among adults aged 65 years and older [9] (Table 1). Overall, these studies agreed with our results, showing the highest seropositivity among young adults and lowest among 40- to 54-year-olds.

Our study found H3N2v seroconversion rates similar to other studies, with 12% seroconverting in the 19–21 year old age group (the only group for which we had pre-vaccination sera) following vaccination. The study in Canada found seroconversion rates were <15% in all age groups and across all vaccine groups [7]. A study by the Centers for Disease Control and Prevention found similar increases against H3N2v among adults who received TIV, with 13–17% experiencing seroconversion. However, they did not find seroconversion in children aged 6–35 months [9]. Similarly, studies among ferrets have also found limited to no protection to H3N2v among individuals vaccinated with TIV [10].

Our study also had rates of seroconversion to seasonal H3N2 similar to those of previous studies that used live attenuated influenza virus (LAIV) [14]. However, our study had much lower seroconversion for seasonal H3N2 compared with the Canadian study that used TIV. Additional seroconversion studies in other age groups will be important for understanding the full benefit of seasonal vaccine protection for H3N2v and seasonal H3N2 viruses.

This study consisted of US DoD service members who are a highly vaccinated and an overall active and healthy population. Consequently, this study population may be less generalizable to the general public who may have lower immune responses to vaccination due to comorbidities. Additionally, a high proportion of the 19- to 21-year-olds in our study received LAIV (77%). Previous studies have found higher efficacy and effectiveness with LAIV compared to TIV among children, but mixed results among adults [15,16]. However, a previous study comparing seroconversion rates among military recruits found that they were higher for TIV [17]. This may be a result of higher mucosal immune response and lower systemic immune response with the intranasal LAIV vaccine, resulting in low seroconversion [18]. Similarly among the 19- to 21-year-olds in our study, the mean geometric mean titers for H3N2v among the LAIV vaccinated group in our study were smaller (130, 95% CI: 86–174) than the TIV vaccinated group (282, 95% CI: 45–519). This could explain why the seroconversion rate we observed was similar to previous studies that examined response to LAIV.

The two older age groups in this study were presumably vaccinated routinely for many years, whereas the younger group likely had low vaccination rates similar to the general population in the years prior to the study. It is postulated that once a person is exposed to an influenza vaccine, he or she may reach an “antibody ceiling,” and his or her antibody titers will not increase in response to infection [14]. However, it has also been found that prior influenza vaccination may negatively impact future influenza immunological responses [19–23], especially previous vaccination with TIV [23]. This may explain, in part, why we saw very low titers against H3N2 (Perth) in the two older age groups compared with the previous US study [9], which likely had lower vaccination rates in prior seasons.

Although serologic testing is often used to identify subclinical infections and infections that would not be identified by real-time polymerase chain reaction, it can be nonspecific, with serologic testing sometimes picking up cross-reacting antibodies from similar influenza strains [24]. Our study found a moderate but significant correlation between H3N2v and seasonal H3N2v geometric mean titers, both before and after influenza vaccination, which may reflect some cross protective immunity between the two virus strains.

Specificity of diagnostic tests can also vary across age groups; one study found lower MN specificity for pH1N1 in older age groups and 80% sensitivity when the ≥80 cutoff was used [25]. Another study found that an HI titer of 110 instead of 40 corresponded to 50% clinical protection among children, therefore different cutoffs should be used for different age groups [26]. Consequently, seroprotection may be overestimated in our young adult group. Additionally, antibody titers decrease over time since vaccination, with faster declines seen among older age groups [27]. These declines were seen as early as 6 months [27] and could have also played a role in the lower titers seen among the two older groups who were sampled on average further from the time of vaccination compared with the youngest group.

Despite these concerns, MN titers measure the number of neutralizing antibodies and may have greater sensitivity than HI antibody titers, especially with novel influenza viruses [25]. Although, the 50% seroprotective level for MN is not known for H3N2v specifically, a previous study found that MN titers were usually double that of HI titers for pH1N1 [25], and previous H3N2v serology studies have used MN and HI cutoffs of ≥80 or ≥40, respectively, to represent a 50% seroprotective level [7,9]. However, the US National Health and Nutrition Examination Survey study, which used both HI and MN titers on the same samples, showed that the percentage with seroprotective titers was consistently higher for MN than HI when the ≥80 and ≥40 cutoffs were used [9]. The variation among and between different serology tests should be taken into consideration when comparing serological results.

Improving our knowledge of H3N2v cross-protective antibodies among specific age groups is important in case this strain becomes more easily transmissible from human to human. The main differences across age groups are likely a result of different influenza exposures to circulating strains during childhood, during which time the strongest immune response is mounted, and differences in exposures later in life that boost antibodies [28]. Identifying groups who are at highest risk is important since there are often shortages of vaccines at the beginning of an outbreak or pandemic. Age groups with lower cross-protective antibodies could be targeted to receive priority vaccination, which could potentially reduce the spread of the virus.

## Supporting Information

S1 IRB Institutional Review Board approval. (PDF)

S2 IRB Institutional Review Board completion. (PDF)

S1 Dataset (XLSX)
