# Supplementary material for: Seroprotective Antibodies to 2011 Variant Influenza A(H3N2v) and Seasonal Influenza A(H3N2) among Three Age Groups of US Department of Defense Service Members
Source: PLoS One. 2015 Mar 27;10(3):e0121037. doi: 10.1371/journal.pone.0121037 (PMC4376909; doi:10.1371/journal.pone.0121037)
Supplement: S1 IRB — (PDF) [file pone.0121037.s001.pdf]

APPENDIX D. POSTAPPROVAL DOCUMENTATION

INSTITUTIONAL REVIEW BOARD RECOMMENDATION

PROTOCOL MODIFICATION

Date of Review:	18 July 2013	Protocol Number: NHRC.2013.0025
Title of Research Protocol:	Serologic Immunity to 2011 Swine-like H3N2v Influenza among DoD Service Members	
Approx. dates of Research:	01 May 2013 to 31 Dec 2013	
Principal Investigator:	CDR Gary Brice, Ph.D.	

The Principal Investigator submitted a modification request for a protocol that was previously classified as exempt. The objective of this research project is to determine the level of serologic immunity to the 2011 H3N2v virus among 3 age groups born roughly 15 years apart and to test the hypothesis that older individuals have greater levels of immunity. Sera samples from DoD service members in 3 distinct age ranges will be tested for immunity to the 2011 H3N2v virus. The Naval Health Research Center (NHRC) has sera from more than 200 basic trainees born in 1992-3 that was obtained in 2011. Fifty (50) of these samples were systematically selected for testing and will be supplemented with de-identified Armed Forces Health Surveillance Center (AFHSC) Department of Defense Sera Repository (DoDSR) specimens from service members born in 1982-3 and 1972-3. Fifty (50) samples will be obtained from both age groups, and all samples will be matched to the 1992-3 DOB set by sex and geographic location. De-identified sera samples will be obtained from the DoDSR and sent frozen to study personnel at NHRC. No personal identifiers will be on the samples.

The modification submission requested to: 1) modify the gap in the 3 age groups to 15 years instead of 10 years to cover a wider range; 2) remove the plan to save any unused sera. All sera will be destroyed after testing is complete; 3) specify the volume of sera in each sample; and 4) clarify the geographic criteria for the DoDSR samples.

The Chair reviewed this modification under the expedited review authority subdelegated by the Naval Health Research Center Commanding Officer and permitted under 32 CFR § 219.110. This protocol is eligible for this type of review under 32 CFR § 219.110(b)(2). The Chair recommends approval of these modifications.

The current IRB approval period expires on 16 June 2014.

Jay H. Heaney, M.A.
Chair, NHRC IRB

Jay H. Heaney 18 Jul 13
Signature & Date

****

DETERMINATION OF APPROVING AUTHORITY

1. I concur with the recommendation of the IRB, and I approve this modification.

   Next review is required no later than: 16 June 2014

2. I concur with the recommendations of the IRB, but I require additional modifications or restrictions prior to providing continuing approval (Attach modifications or restrictions required).

   Next review is required no later than:

3. I disagree with the recommendations of the IRB and recommend (Attach statement regarding recommendations and reasons).

	Signature	Date (MM/DD/YY)
LANNY L. BOSWELL, CAPT, MSC, USN<br>Commanding Officer	[signature]	07/22/13

RECEIVED JUL 0 8 2013

HUMAN USE PROTOCOL
ROUTING SLIP - MODIFICATION

FROM (Principal Investigator):
DR Gary Brice
(TYPED NAME)

(PI SIGNATURE)

In accordance with NAVHLTHRSCHCENINST 3900.2F, I am submitting the attached human use protocol for consideration.

TITLE OF PROTOCOL: Serologic Immunity to 2011 Swine-like H3N2v Influenza among DoD Service Members

ABBREVIATED TITLE: H3N2v Influenza Serology

PROPOSED DATES OF RESEARCH: 1 May 2013 to 31 Dec 2013

SUBMISSION (CHECK ONE):
- ☐ INITIAL SUBMISSION
- ☒ MODIFICATION OF PREVIOUS SUBMISSION
- ☐ CONTINUING/ANNUAL REVIEW

PROTOCOL OBJECTIVE (Brief sentence or two): Determine the level of serologic immunity to the 2011 H3N2v virus among 3 age groups born roughly 15 years apart.

COMMENTS (e.g., issues, special considerations):

DoD PROTOCOL NUMBER: NHRC.2013.0025

Note: You must obtain signatures 1-3 before you submit the protocol to the IRB.		DATE RECEIVED	DATE APPROVED	INITIALS
1	PROGRAM MANAGER	26 June 2013	26 June 2013	
2	PROGRAM IRB MEMBER	" "	" "	
3	DIRECTOR OF SCIENCE AND TECHNOLOGY		3 Jul 13	
4	IRB ADMINISTRATOR	JUL 08 2013		
5	COMMANDING OFFICER			

26 JUN 2013

From: CDR Gary Brice
To: Chair, Institutional Review Board, Naval Health Research Center, San Diego, CA

Subj: MODIFICATION TO PROTOCOL #NHRC.2013.0025, Serologic Immunity to 2011 Swine-like H3N2v Influenza among DoD Service Members

Ref: (a) NAVHLTHRSCHCENINST 3900.2F

1. This modification request is submitted to fulfill the reference (a) requirement for the Principal Investigator to seek approval for any changes that the PI wishes to incorporate to an approved protocol.

2. I certify that all study personnel have been informed of and accept the proposed changes.

3. Point of contact for further information is Tony Hawksworth, anthony.hawksworth@med.navy.mil, (619) 553-7607

26 June 2013

Gary Brice 26 June2013
PI Signature and Date

MODIFICATION OF IRB PROTOCOL

1. **PROTOCOL NUMBER:** NHRC.2013.0025

2. **PROTOCOL TITLE:** Serologic Immunity to 2011 Swine-like H3N2v Influenza among DoD Service Members

3. **WORK UNIT TITLE AND NUMBER:** 60805 - GEIS AI/PI and FRI

4. **PRINCIPAL INVESTIGATOR(S):** CDR Gary Brice, Anthony Hawksworth

5. **RISK LEVEL:** Minimal

6. **CURRENT STUDY STATUS:**

Study has not begun.

7. **CHANGES SINCE LAST REVIEW:**

A, Upon further literature review, we have changed the gap in the 3 age groups to 15 years in order to cover a wider range. This will allow us to test age groups that have shown marked differences in the proportion of individuals to immunity to H3N2v.

B. Upon the suggestions of the DoDSR review group, we have made the following modifications:

1. Dropped the plan to save any unused sera. All sera will be destroyed after testing is complete.
2. Specified the volume of sera in each sample
3. Clarified the geographic criteria for the DoDSR samples – stationed in the US at time of sampling.

PI Signature and Date

DESCRIPTION OF PROPOSED RESEARCH
Serologic Immunity to 2011 Swine-like H3N2v Influenza among DoD Service Members

Research Objective: Recent isolation of a novel swine-origin influenza A H3N2 variant virus (H3N2v) from humans in the United States has raised concern over the pandemic potential of these viruses. The objective of this study is to determine the level of serologic immunity to the 2011 H3N2v virus among 3 age groups born roughly ~~10~~ 15 years apart and to test the hypothesis that older individuals have greater levels of immunity. Previous studies have suggested that large differences in the proportion with serologic immunity to H3N2v exist between age groups (CDC, MMWR, April 2012; Waalen et. al., Euro Surveill., May 2012). Our proposed sample size of 50 in each age group will allow us to detect similar differences in seroprevalence with 95% confidence.

Research Procedures: Under this proposal, sera samples from DoD service members in 3 distinct age ranges will be tested for immunity to the 2011 H3N2v virus. NHRC has sera from more than 200 basic trainees born in 1992-3 that was obtained in 2011. Fifty of these samples were systematically selected for testing and will be supplemented with de-identified Armed Forces Health Surveillance Center Department of Defense Sera Repository (DoDSR) specimens from service members born in ~~1982-3~~ 1977-8and ~~1972-3~~1962-3. Fifty samples will be obtained from both age groups, and all samples will be matched to the 1992-3 DOB set by sex and geographic location (stationed in US at time of sampling).

De-identified sera samples with a volume of 0.3 – 0.5 mil each will be obtained from the DoDSR and sent frozen to Mr. Hawksworth at NHRC. Each specimen will arrive labeled with a unique ID that will not contain any PHI. There will be no attempt to "link-back" the specimens to the individuals. We are requesting 2 sets of 50 samples; 1 set from 30 year old service members (at the time of sampling) and 1 set from 40 year olds (at the time of sampling). Each set of 50 will meet the following criteria:

- Collected between 1/1/2011 and 6/30/2011
- Stationed in the US at time of collection
- Matched by sex with a set of 2011 samples from 20 year olds already at NHRC (see attached list)

Testing will be performed with the MN assay using established, validated techniques. The H3N2v virus and the corresponding antisera will be obtained from US Centers for Disease Control collaborators. A serologic MN titer of >= 1:80 will be considered protective. Data will be reported in aggregate and there will be no attempt to "link-back" the specimens to the individuals.

NHRC will ~~keep the untested remainder of specimens should similar questions for a different pathogen arise in the next few years~~destroy any remaining sera samples after testing and analyses are complete. ~~Specimens will remain de-identified and stored securely at -70C. IRB approval will be sought for any future studies that would utilize these specimens.~~

Risks to Participants: None. All specimens will be de-identified. The specimens used in this research were previously collected, thus active enrollment/recruitment of subjects will not take place. This study will use existing serum samples from the DoDSR that have been collected as a part of ongoing DoD testing programs. Thus, the study poses no physical risks to the participants. There is no risk of study subjects being identified or linked to their specimen.

Means of Protecting Against Risks: All specimens will be de-identified. Data will be maintained on password-protected computers on secure servers and only approved investigators will have access to the data. All investigators will have completed HIPAA training prior to the start of the study.

Conflict of Interest: No person involved in the design, conduct, or reporting of this research has a financial or other interest that could possibly appear to be affected by the carrying out o results of this research.

Informed Consent: A Waiver of the Consent is requested as:

- this research does not involve more than minimal risk to the subjects
- the waiver or alteration will not adversely affect the rights and welfare of the subjects
- the research could not practicably be carried out without the waiver or alteration
