# Supplementary material for: Seroprotective Antibodies to 2011 Variant Influenza A(H3N2v) and Seasonal Influenza A(H3N2) among Three Age Groups of US Department of Defense Service Members
Source: PLoS One. 2015 Mar 27;10(3):e0121037. doi: 10.1371/journal.pone.0121037 (PMC4376909; doi:10.1371/journal.pone.0121037)
Supplement: S2 IRB — (PDF) [file pone.0121037.s002.pdf]

INSTITUTIONAL REVIEW BOARD RECOMMENDATION

**COMPLETION REPORT**

Date of Review: 11 June 2014 Protocol Number: NHRC.2013.0025

Protocol Title: Serologic Immunity to 2011 Swine-like H3N2v Influenza among DoD Service Members

Principal Investigator: CDR Gary Brice, Ph.D.

The Principal Investigator submitted a completion report for a protocol that was previously classified as minimal risk. The objective of this research project was to determine the level of serologic immunity to the 2011 H3N2v virus among 3 age groups born roughly 15 years apart and to test the hypothesis that older individuals have greater levels of immunity. Sera samples from DoD service members in 3 distinct age ranges were tested for immunity to the 2011 H3N2v virus. Fifty sera samples of recruits born in 1992-93 held at NHRC were systematically selected for testing and were supplemented with de-identified Armed Forces Health Surveillance Center (AFHSC) Department of Defense Sera Repository (DODSR) specimens from service members born in 1982-83 and 1972-73. The samples obtained from AFHSC were matched to the 1992-93 birth year data set by sex and geographic location and analyzed using microneutralization assays.

The results obtained are similar to previous studies which have found the highest sero-protection among young adults and decreasing titers among older adults. The proportion of 19-21 year olds who seroconverted after seasonal vaccination was low and similar to previous findings. Improving the understanding of H3N2v immunity among different age groups can help inform vaccination plans if H3N2v becomes more transmissible in the future.

I recommend forwarding the completion report to DON HRPP.

| | Signature | Date |
|---|---|---|
| Institutional Review Board Chair<br>Naval Health Research Center | Christopher G. Blood, JD, MA | 6/11/14 |
| Commanding Officer<br>Naval Health Research Center | J.D. RYCHNOVSKY, CAPT, NC, USN | 6/12/14 |

RECEIVED JUN 0 9 2014

9 June 2014

From: CDR Gary Brice, MSC, USN, Head, Operational Infectious Diseases Department, Naval Health Research Center, San Diego, CA
To: Chair, Institutional Review Board, Naval Health Research Center, San Diego, CA

Subj: COMPLETION REPORT FOR PROTOCOL # NHRC.2013.0025; Serologic Immunity to 2011 Swine-like H3N2v Influenza among DoD Service Members

Ref: (a) NAVHLTHRSCHCENINST 3900.2F

Encl: (1) Completion Report for Protocol #NHRC.2013.0025

1. Enclosure (1) is submitted to fulfill the reference (a) requirement for a completion report for the subject research protocol.

2. Point of contact for further information is Mr. Tony Hawksworth, anthony.hawksworth@med.navy.mil, 619-553-7607.

9 June 2014

CDR Gary Brice
PI Signature and Date

# COMPLETION REPORT FOR IRB PROTOCOL

1. **PROTOCOL NUMBER:** NHRC.2013.0025

2. **PROTOCOL TITLE:** Serologic Immunity to 2011 Swine-like H3N2v Influenza among DoD Service Members

3. **WORK UNIT TITLE AND NUMBER:** 60805; Navy Center of Excellence for Pandemic Influenza Surveillance and Testing; Domestic and Deployed Laboratory Network at the Navy Respiratory Disease Laboratory. Population-based surveillance for Febrile Respiratory Illness & Pneumonia

4. **PRINCIPAL INVESTIGATOR (S):** CDR Gary Brice

5. **OBJECTIVE**
The objective of this study was to determine the level of serologic immunity to the 2011 H3N2v virus among 3 age groups born roughly 15 years apart and to test the hypothesis that older individuals have lower levels of immunity.

6. **APPROACH**
Sera collected in 2011 were obtained from DoD Service Members in three age groups: 19-21 year olds (n=49), 32-33 year olds (n=50), and 47-48 year olds (n=50). Pre- and post-vaccination samples were available for the youngest age group, and only post-vaccination for the older 2 age groups. Specimens were tested using microneutralization (MN) assays for antibody titers against H3N2v (A/Indiana/10/2011) and seasonal H3N2 virus (A/Perth/16/2009). A serologic MN titer of >= 1:80 was considered protective.

7. **RESULTS/FINDINGS**
The youngest age group had significantly ($p<0.05$) higher geometric mean titers for H3N2v with 165 (95% CI: 105, 225), compared to the two oldest age groups, 32-33 and 47-48 year olds, who had geometric mean titers of 68 (95% CI: 55, 82) and 46 (95% CI: 24, 65), respectively. Similarly, the youngest age group also had the highest geometric mean titers for seasonal H3N2. In the youngest age group, the proportion that seroconverted after vaccination was 12% for H3N2v and 27% for seasonal H3N2.

8. **ADVERSE EVENTS**
Not applicable. The samples used in this study were previously collected for other purposes.

9. **CONCLUSIONS**
Our results were similar to previous studies which found highest seroprotection amongst young adults and decreasing titers among older adults. The proportion of 19-21 year olds who seroconverted after seasonal vaccination was low and similar to previous findings. Improving our understanding of H3N2v immunity among different age groups in the United States can help inform vaccination plans if H3N2v becomes more transmissible in the future.

10. **ACCOMPLISHMENTS**

    a. Journal Publications
    b. Technical Reports and Documents
    c. Letter Reports to Fleet Users
    d. Presentations and Abstracts
    e. Patents
    f. CRADAs
    g. Awards
